# The Impact of the Novel Coronavirus Severe Acute Respiratory Syndrome Coronavirus 2 (SARS-CoV-2) Pandemic on Case Fatality Rates and Cost of Surgical Care in Brazil

**DOI:** 10.7759/cureus.56624

**Published:** 2024-03-21

**Authors:** Wilhelmina N Hauwanga, Noama El Husseini, Abdullah A El Ghazzawi, Zaeemah Mansoor, Abhishek Chaudhary, Aisha Elamin, Billy McBenedict

**Affiliations:** 1 Family Medicine, Faculty of Medicine, Federal University of the State of Rio de Janeiro, Rio de Janeiro, BRA; 2 Faculty of Medicine, Beirut Arab University, Beirut, LBN; 3 Faculty of Health Sciences, Karachi Medical and Dental College, Karachi, PAK; 4 Medicine, Smt. Nathiba Hargovandas Lakhmichand Municipal Medical College, Ahmedabad, IND; 5 Faculty of Medicine, National University of Sudan, Khartoum, SDN; 6 Medicine, Hospital Universitário Antônio Pedro (Antonio Pedro University Hospital), Niteroi, BRA

**Keywords:** covid-19, brazil, case fatality rates, sars-cov-2 pandemic, surgical care

## Abstract

Background

The coronavirus disease 2019 (COVID-19) pandemic provoked disruptions in healthcare delivery. Following the recommendations of major surgical societies and surgical departments globally, most surgeries were widely canceled or postponed, causing significant disruptions to healthcare delivery worldwide, including in Brazil. Brazil's public healthcare system - Sistema Único de Saúde (SUS) was particularly affected, with a substantial decline in elective procedures, especially during the pandemic's early stages. The impact of the pandemic on surgical services in Brazil has not been adequately studied since most studies only cover the early phases of the pandemic. Thus, this study aims to analyze the case fatality rates and costs, associated with the different surgical procedure subgroups performed during the pre-pandemic, pandemic, recovery, and post-pandemic periods in all five regions of Brazil.

Methods

A retrospective cross-sectional design was used to examine surgical cases from 2019 to 2022. Data was divided into four time periods, named as the pre-pandemic (March-December 2019), pandemic (March-December 2020), recovery (March-December 2021), and post-pandemic (March-December 2022), and was analyzed for the cost of surgical procedures in the aforementioned four periods. In addition, the case fatality rates and rate ratios in the four periods stratified according to region were calculated.

Results

The cost of surgical procedures decreased during the pandemic and recovery period compared with pre-pandemic for all procedures except thoracic surgery where it was higher in the recovery period than pre-pandemic. No statistically significant change in cost was observed in surgeries of the central and peripheral nervous system, circulatory system, obstetric, and oncology. Case fatality rates increased among all five regions of Brazil in pandemic and recovery periods compared to pre-pandemic and post-pandemic periods. Case fatality rates increased during the pandemic and/or recovery as compared to pre-pandemic in all procedures except visual apparatus and obstetric surgeries were not affected by the pandemic in terms of case fatality rates.

Conclusion

The COVID-19 pandemic had a significant impact on surgical care costs and case fatality rates for surgery in Brazil. There was a decreasing trend in the costs of procedures during the pandemic, followed by a gradual recovery to baseline values, except for thoracic surgery. Case fatality rates rose initially and then declined, ultimately reaching baseline levels. The pandemic posed significant challenges to the healthcare system, affecting medical services, including surgical care.

## Introduction

The COVID-19 pandemic, caused by the novel coronavirus Severe Acute Respiratory Syndrome Coronavirus 2 (SARS-CoV-2), had a profound impact on global health and economies. It led to a major disruption of routine hospital services, and decision-makers had to reallocate hospital resources, including beds, staff, and critical supplies like personal protective equipment, ventilators, and oxygen, towards the COVID-19 response to ensure there were enough resources to fight the pandemic [1].

Surgery, a cornerstone of modern medicine, has always carried inherent risks, with mortality being a critical measure of its safety and success. However, the emergence of the novel coronavirus introduced a complex array of challenges with the potential to alter this long-standing metric. The convergence of strained healthcare resources, and shifting surgical interventions during the pandemic. Most of the large surgical societies worldwide and surgical departments globally implemented elective surgery cancellations to safeguard patients and staff, while also conserving resources in anticipation of COVID-19 case surges [2].

The COVID-19 pandemic had a substantial financial impact on the surgical specialty because elective surgeries are a major source of revenue for hospitals, often constituting up to two-thirds of their income [3]. Due to the high load of COVID patients and less availability of general beds, most surgical wards were converted to COVID units to increase the bed capacity [3]. In addition, there were also many cancellations of surgical cases due to pre-procedural positive COVID-19 testing results which led to a time delay in surgeries and eventually piling of cases [3]. This ultimately led to the cancellation of non-urgent procedures and major financial setbacks for most surgical departments and the overall healthcare system.

COVIDSurg Collaborative estimated that about 28 million surgeries were canceled or postponed globally during the peak 12 weeks of the COVID-19 pandemic [4]. This trend was also seen in Brazil, a country geographically divided into five regions: North, Northeast, Southeast, South, and Midwest [5]. In Brazil, COVID-19 caused a significant reduction in elective procedures in the public healthcare system - Sistema Único de Saúde (SUS). The reduction was most pronounced in the first 9 months of the pandemic (April-December 2020), with a total of 828,429 elective procedures canceled or postponed [6]. As COVID-19 has had devastating effects in Brazil, approaches that explore differences between regions and states are vital for identifying factors associated with the heterogeneous behavior of the disease from a population perspective.

There have not been studies that have comprehensively recorded the effect of the COVID-19 pandemic on the Brazilian healthcare system. This is desirable because such studies serve as a basis for interventions should a similar incidence (pandemic) occur in the future. Our study aimed to provide an in-depth analysis and study of the effect of COVID-19 on the case fatality rate (CFR) and costs of surgical procedures in Brazil. Analyzing its impact on surgical care helps identify how the Brazilian system responded and provides a road map for future interventions and preparedness strategies in case pandemics occur in the future. In addition, any disruptions or alterations to surgical services can have profound implications for patient health and well-being. This is the second part of our article series regarding the impact of the COVID-19 pandemic on the Brazilian healthcare system. Brazil is among the best countries to carry out this type of study as it is one of the countries that was affected the most by the COVID-19 pandemic. Surgeries of low and medium complexity, transplants, and screening procedures were the most affected [7]. We analyzed data at the regional level and this is of importance as Brazil is one of the countries with the highest level of inequalities [6,8], for example, the South and Southeast regions exhibit a greater concentration of healthcare workers including surgeons, anesthesiologists, and obstetricians, compared to the North and Northeast regions, which are considered the most economically disadvantaged areas in Brazil [9]. The rationale of the study was to analyze the impact of the pandemic on surgical care at a regional level and allow the identification of differences in response and outcomes across various areas in Brazil. This analysis is crucial for providing a basis for tailoring region-specific interventions and allocating resources equitably in the face of healthcare inequalities. We also analyzed the (1) costs of procedures according to subgroups of procedures performed during the pre-pandemic, pandemic, recovery, and post-pandemic periods, (2) case fatality rates and rate ratios in the four periods stratified according to the region along with (3) case fatality and rate ratios for procedure subgroups for the years 2019-2022.

## Materials and methods

Study design

This study used a retrospective cross-sectional study design. A total of 15 subgroup surgical procedures from DATASUS were considered for analysis in this study. The data was collected from various hospitals in Brazil at the national level, and analysis was made at national and regional levels. The data was divided into four categories denoting four time periods to allow for better resolution in comparison. The four periods were pre-pandemic (March-December 2019), pandemic (March-December 2020), recovery (March-December 2021), and post-pandemic (March-December 2022). Data from the same months (March-December) for each year was deliberately used for consistency and ease of comparison. The pre-pandemic data was used as the baseline data for analysis in this study.

Data collection

Data was collected from the Brazilian Hospital Information System records under the “Departamento de Informática do SUS (DATASUS),” which is housed by the Sistema Único de Saúde (SUS), a Brazilian Unified Health System. DATASUS is the Brazilian Public Unified Health System (SUS) department, it provides health data to be used by several studies conducted across the country. Furthermore, it is a primary source of research data for the field of surgery and surgical subspecialties [10]. Regarding data quality and completeness, the data used had no missing values. This system encompasses data from all hospitalizations reimbursed by SUS, covering about 80% of the population. It stands as a testament to Brazil’s successful experience in the domain of health information management, a project spearheaded by the Federal Government of Brazil. DATASUS accumulates information from both public and private institutions affiliated with SUS and is responsible for practical implementation of research in the health area after incorporating and analyzing the information [10].

Inclusion and exclusion criteria

The inclusion criteria were the cost of various surgical procedures and number of deaths occurring in each subgroup of surgical cases performed during the aforementioned four periods (pre-pandemic, pandemic, recovery, and post-pandemic). The subgroups of surgical cases included in this study are “surgery of the musculoskeletal system,” “surgery of the visual apparatus,” “surgery of the digestive system,” “associated organs and abdominal wall,” “surgery of the genitourinary system,” “thoracic surgery,” “oral and maxillofacial surgery,” “surgeries of the skin,” “subcutaneous tissue and mucosa,” “endocrine gland surgery,” “breast surgery,” “central and peripheral nervous system surgery,” “upper airway, face,” “head and neck surgery,” “circulatory system surgery,” “obstetric surgery,” “reconstructive surgery,” and “surgery in oncology”. To standardize the study periods for comparison, surgical procedures conducted in the months of January and February (2019-2022) were excluded. This decision was made because the pandemic only became apparent in Brazil in March 2019.

Data analysis

Costs for Surgical Procedures

The costs for surgical procedures in the four periods stratified according to the subgroup were presented using summary statistics (percentages). Data was analyzed for the costs of 15 surgical procedures based on subgroups performed during the pre-pandemic, pandemic, recovery, and post-pandemic periods. The total cost for each subgroup procedure was compared across the pre-pandemic (March-December 2019), pandemic (March-December 2020), recovery (March-December 2021), and post-pandemic (March-December 2022) by calculating the percentage of the cost of each subgroup procedure for each year based on the total cost for the study period, for example the percentage for the cost of surgery of the musculoskeletal system was calculated as follows.

(7,533,105,836/27,449,968,554) *100 = 27.4% for pre-pandemic, 

(6,241,898,131/27,449,968,554) *100 = 22.7% for pandemic, 

(6128450821/27,449,968,554) *100 = 22.3% for the recovery, and (7,546,513,766/27,449,968,554) * 100 = 27.5% for post-pandemic.

To assess if the observed differences were statistically significant, further data analysis was performed using Statistical Package for the Social Sciences (SPSS) version 27 (IBM SPSS Statistics, Armonk, NY, USA) as outlined in the literature [11]. A normality test was done using the Kolmogorov-Smirnov test, and non-normally distributed data was analyzed using the Kruskal-Wallis test to assess whether the differences were statistically significant across the four periods. For the same reason, a one-way analysis of variance (ANOVA) test was used for normally distributed data.

Case Fatality Rates

In addition, the case fatality rates and rate ratios in the four periods were calculated according to region and type of surgery. The CFR is a measure that corresponds to the proportion of individuals with a specific condition who died from that condition during the reference period. In our study, the CFR was calculated as the proportion of people who died following surgery over the total number of people admitted to the hospital for surgery during four time frames. For example, the CFR for the North region was calculated as the proportion of people who died following surgery in the North region over the total number of people admitted to the hospital for surgery during four time frames in all the regions. The same was done for the surgery subgroups but only that the factor of interest was the surgery subgroup and not the region. Rate ratios were calculated by using the pre-pandemic period as the reference year, and by dividing the CFR for the pandemic, recovery, and post-pandemic by the pre-pandemic.

## Results

Costs of procedures according to subgroups of procedures

A comprehensive analysis of procedure costs, categorized by subgroups, and examined across distinct temporal periods revealed no statistically significant differences in procedure costs within the subgroups of “central and peripheral nervous system surgery,” “upper airway, face, head and neck surgery,” “circulatory system surgery,” “obstetric surgery,” “reconstructive surgery,” and “surgery in oncology” during the various periods as indicated in Table 1. Conversely, notable variation in costs was observed for other types of surgical procedures across the pre-pandemic, pandemic, recovery, and post-pandemic phases. These procedures exhibited higher costs in the pre-pandemic period, underwent a substantial decline during the pandemic, and subsequently demonstrated a gradual reversion towards baseline levels during the recovery and post-pandemic periods as seen in “Surgery of the Musculoskeletal System”. Procedures that exceeded the baseline cost are “surgery of the visual apparatus,” “surgery of the digestive system, associated organs and abdominal wall,” “surgery of the genitourinary system,” “thoracic surgery,” and “oral and maxillofacial surgery”. Procedure costs that decreased during the pandemic and recovery period, followed by an increase yet not reaching the pre-pandemic values are “surgeries of the skin, subcutaneous tissue and mucosa,” “endocrine gland surgery,” and “breast surgery”. The differences in costs within these subgroups were statistically significant.

**Table 1:** Costs of procedures according to subgroups of procedures performed during the pre-pandemic, pandemic, recovery, and post-pandemic periods. The currency of the costs is Brazilian Real.

Procedure Subgroup	Pre-pandemic 2019 N (%)	Pandemic 2020 N (%)	Recovery 2021 N (%)	Post-pandemic 2022 N (%)	p-value
Surgery of the musculoskeletal system	7,533,105,836 (27.4)	6,241,898,131 (22.7)	6,128,450,821 (22.3)	7,546,513,766 (27.5)	0.001
Surgery of the visual apparatus	131,104,503 (28.8)	7,786,877,115 (17.1)	9,584,152,027 (21.1)	1,500,186,952 (33.0)	0.002
Surgery of the digestive system, associated organs and abdominal wall	6,942,586,805 (29.6)	4,408,490,427 (18.8)	4,721,266,193 (20.1)	740,797,273 (31.5)	0.004
Surgery of the genitourinary system	2,564,196,091 (29.0)	1,498,421,127 (17.0)	1,754,579,642 (19.9)	3,021,207,594 (34.2)	0.000
Thoracic surgery	1,527,453,658 (22.7)	1,554,450,165 (23.1)	1,870,870,794 (27.8)	1,765,389,775 (26.3)	0.000
Oral and maxillofacial surgery	529,980,406 (28.2)	284,410,478 (15.1)	394,301,608 (21.0)	672,126,336 (35.7)	0.000
Surgeries of the skin, subcutaneous tissue, and mucosa	358,452,137 (31.8)	2,163,104,958 (19.2)	2219,107,416 (19.7)	3,322,014,301 (29.4)	0.002
Endocrine gland surgery	698,275,038 (33.8)	309,711,434 (15.0)	38,422,291 (18.6)	671,485,528 (32.5)	0.001
Breast surgery	1,681,842,977 (36.4)	757,618,652 (16.4)	878,811,682 (19.0)	1,302,910,722 (28.2)	0.000
Central and peripheral nervous system surgery	2,562,389,786 (26.9)	2,317,274,601 (24.3)	2,183,442,073 (22.9)	2,473,069,583 (25.9)	0.317
Upper airway, face, head and neck surgery	1,737,881,786 (27.8)	1,318,362,935 (21.1)	1,687,231,867 (27.0)	1,514,494,639 (24.2)	0.009
Circulatory system surgery	1,487,467,683 (28.1)	1,221,868,525 (23.0)	1,212,109,989 (22.9)	1,379,739,382 (26.0)	0.676
Obstetric surgery	6,449,918,585 (26.0)	6,435,108,514 (25.9)	5,935,941,494 (23.9)	5,998,350,545 (24.2)	0.176
Reconstructive surgery	7,129,751,723 (27.7)	6,104,788,898 (23.7)	5,773,185,959 (22.4)	6,744,005,148 (26.2)	0.006
Surgery in oncology	4,500,934,114 (26.9)	3,931,511,114 (23.5)	3,884,920,696 (23.3)	4,391,914,755 (26.3)	0.072

Case fatality rate and rate ratios according to region

The case fatality rates and rate ratios for different regions for the pre-pandemic, pandemic, recovery, and post-pandemic periods were calculated and are presented in Table 2. Among all five regions of Brazil, there was a noticeable trend: the case fatality rates increased in the pandemic (2020) and recovery (2021) period followed by a return to slightly below the baseline (2019) in the post-pandemic period (2022).

**Table 2:** The case fatality rates and rate ratios for the four study periods, stratified according to region of residency.

	Pre-pandemic	Pandemic		Recovery		Post-pandemic	
Regions	Case fatality rate	Case fatality rate	Rate ratio	Case fatality rate	Rate ratio	Case fatality rate	Rate ratio
North	0.0117	0.0123	1.0515	0.0127	1.0802	0.0101	0.8651
North East	0.0128	0.0158	1.2309	0.0158	1.2324	0.0122	0.9485
Southeast	0.0172	0.0216	1.2531	0.0224	1.2998	0.0163	0.9451
South	0.0189	0.0240	1.2726	0.0261	1.3842	0.0184	0.9765
Midwest	0.0135	0.0164	1.2114	0.0160	1.1864	0.0128	0.9464

Case fatality rate and ratios according to surgery type

Procedures including “central and peripheral nervous system surgery,” surgery of the digestive system, associated organs and abdominal wall,” “surgery of the musculoskeletal system,” “reconstructive surgery,” and “surgery in oncology” exhibited a similar trend (Table 3), with a marked increase in CFR ratio during the pandemic, followed by a considerable progressive decrease during the recovery, and post-pandemic periods. “Surgeries of the skin, subcutaneous tissue and mucosa,” “endocrine gland surgery,” “upper airway, face, head and neck surgery,” “circulatory system surgery,” “surgery of the genitourinary system,” “obstetric surgery,” and “thoracic surgery” exhibited a comparable pattern, characterized by a progressive increase in the CFR ratio during the pandemic, and the recovery period. However, during the post-pandemic period, the CFR ratio decreased. “Surgery of the visual apparatus” and “breast surgery” were not affected by the pandemic, as evidenced by the CFR values (Table 3).

**Table 3:** Case fatality rates and respective rate ratios for the procedure subgroups for the years 2019-2022.

	Pre-pandemic	Pandemic		Recovery		Post-pandemic	
Procedure subgroup	Case fatality rate (95% CI)	Case fatality rate (95% CI)	Rate ratio	Case fatality rate (95% CI)	Rate ratio	Case fatality rate (95% CI)	Rate ratio
Central and peripheral nervous system surgery	0.0770 (0.0752-0.0788)	0.0969 (0.0946-0.0992)	1.2584	0.0954 (0.0933-0.0977)	1.2390	0.0714 (0.0696-0.0732)	0.9273
Surgery of the digestive system, associated organs and abdominal wall	0.0183 (0.0180-0.0186)	0.0259 (0.0255-0.0263)	1.4153	0.0256(0.0252-0.0260)	1.3989	0.0164 (0.0161-0.0166)	0.8962
Surgery of the musculoskeletal system	0.0097 (0.0095-0.0099)	0.0112 (0.0110-0.0115)	1.1546	0.0111 (0.0108-0.0112)	1.1443	0.0093 (0.0091-0.0095)	0.9588
Reconstructive surgery	0.0135 (0.0126-0.0145)	0.0181 (0.0169-0.0193)	1.3407	0.0180 (0.0168-0.0193)	1.3333	0.0151 (0.0141-0.0163)	1.1185
Surgery in oncology	0.0164 (0.0158-0.0170)	0.0180 (0.0173-0.0187)	1.0976	0.0172 (0.0165-0.0178)	1.0488	0.0134 (0.0129-0.0140)	0.8171
Surgeries of the skin, subcutaneous tissue and mucosa	0.0010 ( 0.0008-0.0012)	0.0014 (0.0012-0.0017)	1.4000	0.0015 (0.0012-0.0018)	1.5000	0.0009 (0.0007-0.0011)	0.9000
Endocrine gland surgery	0.0014 (0.0008-0.0022)	0.0026 (0.0015-0.0042)	1.8571	0.0028 (0.0017-0.0043)	2.0000	0.0020 (0.0013-0.0031)	1.4286
Upper airway, face, head, and neck surgery	0.0312 (0.0302-0.0321)	0.0491 (0.0476-0 .0506)	1.5737	0.0551 (0 .05360-0.0567)	1.7660	0.0252 (0.0243-0.0262)	0.8077
Circulatory system surgery	0.0292 (0.0286-0 .0298)	0.0365 (0.0357-0.0373)	1.2500	0.0373 (0.0365-00381)	1.2774	0.0294 (0.0287-0.0300)	1.0068
Surgery of the genitourinary system	0.0031 (0.0030-0.0033)	0.0045 (0.0043-.0048)	1.4516	0.0048 (0.0045-0.0050)	1.5484	0.0027 (0.0026-0.0029)	0.8710
Obstetric surgery	0.0004 (0.0004-0.0005)	0.0005 (0.0005-0.0005)	1.2500	0.0006 (0.0006-0.0007)	1.5000	0.0004 (0.0004-0.0004)	1.0000
Thoracic surgery	0.1277 (0.1249-0.1306)	0.1530 (0.1498-0.1563)	1.1981	0.1731 (0.1698-0.1764)	1.3555	0.1252 (0.1224-0.1280)	0.9804
Oral and maxillofacial surgery	0.0014 (0.0009-0.0021)	0.0034 (0.0022-0.0049)	2.4286	0.0061 (0.0047-0.0078)	4.3571	0.0036 (0.0028-0.0047)	2.5714
Surgery of the visual apparatus	0.0002 (0.0001-0.003)	0.0001 (0.0000-0.002)	0.5000	0.0002 (0.0001-0.003)	1.0000	0.0002 (0.0002-0.0003)	1.0000
Breast surgery	0.0004 (0.0025-0.0007)	0.0002 (0.0001-0.0006)	0.5000	0.0004 (0.0002-0.0007)	1.0000	0.0004 (0.0002-0.0007)	1.0000

## Discussion

This study analyzed the case fatality rates, and costs, associated with the different surgical procedure subgroups performed during the pre-pandemic, pandemic, recovery, and post-pandemic periods in all five regions of Brazil. The COVID-19 pandemic had a significant impact on surgical practices worldwide due to case prioritization, operative techniques, and workforce planning [12]. Elective surgeries were postponed and caused significant financial setbacks to hospitals.

Costs of procedures

The costs of procedures according to subgroups of procedures performed during the pre-pandemic, pandemic, recovery, and post-pandemic periods were analyzed and most procedures exhibited higher costs in the pre-pandemic period, underwent a substantial decline during the pandemic, and subsequently demonstrated a gradual reversion towards baseline levels during the recovery and post-pandemic periods. The decreased cost or money spent on different surgeries during the pandemic can be attributed to the reduced volume of surgical procedures performed, especially for elective surgeries. Another possible explanation for the low cost observed during the pandemic is that resources, including financial and manpower, were being redirected towards fighting COVID-19. A significant portion of hospital revenue is derived from surgical procedures and outpatient consultations. Hence, the cancellation of costly procedures and the deferment of elective surgeries can result in a depletion of resources, exacerbating the existing financial crisis [13]. During the pandemic, a higher budget was needed for ventilators, equipment for personnel protection, and hospital staff [14].

Death rates and rate ratios based on regions

Data analyzed per different Brazilian regions showed that the CFR and rate ratios increased during the pandemic, decreased during the recovery period, and decreased even further during the postoperative period. This trend was observed in all regions. However, the Northern region was the least affected, which is likely explained by the lesser surgical load compared to other regions in Brazil. Studies have shown that the Northern region had the second lowest number of surgery admissions compared to other regions in Brazil [11], and the greatest decrease in both emergency and elective neurosurgical cases compared to the other regions [15]. Notably, the COVID-19 pandemic significantly impacted mortality rates among surgical patients in the Southeast and South regions of Brazil, with a higher increase in the case fatality rate. This could be explained by the fact that states in the Southeast region, especially São Paulo and Rio de Janeiro, were the starting point of the COVID-19 pandemic in Brazil [16]. The strain on healthcare resources and the high prevalence of COVID-19 likely led to higher mortality rates among surgical patients during the pandemic. Many of the hospitals in this region postponed or canceled elective surgeries to focus on COVID-19 care and conserve resources during the pandemic [11]. This reduction may have shifted surgical caseloads towards more urgent or emergent procedures with higher mortality rates, contributing to increased surgical mortality during that period [15].

Case fatality rate and rate ratios of surgical procedures

Our findings revealed that the majority of surgical subgroups examined experienced a significant surge in both the death rate and rate ratio during the pandemic. This was followed by a notable gradual decline, towards baseline levels in the recovery and post-pandemic periods. This is in agreement with another study [17], which found that postoperative mortality is almost 6 times higher for patients infected with COVID-19 within 2 weeks of surgery. The increase in the case fatality rates and rate ratios during the COVID-19 pandemic can be attributed to many factors including but not limited to; lack of resources, decreased care for surgical patients during the pandemic, and postoperative complications. A study conducted at several hospitals in Wuhan in 2020 on clinical characteristics and outcomes of patients undergoing surgeries during the incubation period of COVID-19 infection showed that 44·1% of patients needed ICU care, and mortality was 20·5% [18]. Patients undergoing surgery during the COVID-19 pandemic were at risk of hospital-acquired COVID-19 infection and postoperative complications due to pro-inflammatory cytokines, immunosuppressive response to surgery, and mechanical ventilation [18].

COVID-19 patients developed an immediate impairment of immune function during surgical procedures, triggering a defense response known as Systemic Inflammatory Response [19]. This compromised immune system, combined with various other factors, enables the virus to exert greater influence, consequently leading to elevated levels of mortality. In addition, patients diagnosed with COVID-19 either at the time of surgery or within 30 days of surgery face a 7.9 times higher risk of perioperative death [17].

Another possible explanation for the increased surgical CFR during the pandemic is the poor control of patients’ comorbidities, as reported in a previous study [20]. Chronic illnesses such as hypertension and diabetes need regular check-ups to ensure these diseases are controlled. The pandemic disrupted a lot of checkups due to patients fearing that they would acquire COVID-19 from the hospital [17]. In addition, many facilities postponed/canceled appointments during the pandemic. If these patients ended up needing emergency surgery, there would be a much higher risk of mortality. Studies done on hypertensive patients undergoing surgery concluded that: (i) treatment of hypertension should be maintained throughout the perioperative period, and (ii) untreated hypertensive patients should be treated before elective surgery under the impression that greater hemodynamic stability would reduce complications [21]. Furthermore, the observed patterns in surgical case fatality rates could be attributed to the imposed limitations on allowing family members to accompany patients. As a result, numerous patients may have opted to cancel their appointments rather than face the prospect of being isolated from their crucial support system during their most critical moments of need. A study identified two major factors associated with worse perioperative outcomes: emotional vulnerability, and surgery in hospitals with SARS-CoV2 wards [22]. Family support is a strong mediator for the development of resilience in various disease states. All the aforementioned reasons can also be attributed to the decrease in CFR and rate ratios towards baseline during the recovery and post-pandemic period. The results of this study showed that there was an increase in mortality during the pandemic period and/or recovery time as compared to pre-pandemic. The exceptions were the visual apparatus and obstetric surgery, which were not affected by the pandemic.

Our study offers a detailed overview of how a pandemic, such as COVID-19, impacts the healthcare system of upper-middle-income countries like Brazil. The conclusions drawn from our study can provide valuable insights for other middle-income countries like Brazil, to take appropriate steps in the future. Pandemics create crises that require timely measures from governments to optimize the delivery of healthcare services. Brazil’s diversity and significant regional differences were also highlighted in this study with respect to responses to the COVID-19 pandemic. Understanding and addressing these regional differences is crucial for an effective pandemic response and healthcare system optimization in Brazil. To strengthen surgical care delivery during pandemics, several areas need to be addressed. Firstly, clear guidelines should be implemented within the Brazilian healthcare system to prioritize surgeries without compromising patients' health. Secondly, investing in training programs for medical personnel who provide surgical care in disaster medicine and pandemics can better equip healthcare workers to handle crises effectively. Thirdly, integrating telemedicine into routine care delivery can mitigate disruptions caused by pandemics, enabling continued care for chronic illnesses, and reducing the likelihood of complications and the need for emergency surgeries. Lastly, encouraging regular health check-ups to assess the population's well-being, especially for chronic diseases, is essential. Apart from these guidelines, many studies have suggested different ways to cope with this situation. Since the cancellation of elective surgeries in the early phases of the pandemic created a backlog, suggested ways of clearing this backlog include longer working days and working weekends [23]. Furthermore, the Medically Necessary Time-Sensitive Procedure (MeNTS) scoring system - is a useful tool for case priority allocation when recommencing elective surgery. This scoring system enables surgeons to triage elective surgery cases objectively and reduces the ethical burden [24].

Limitations and strengths

This study presents certain limitations. The data was obtained solely from one country, which might limit the generalization of the results to other nations. Regarding data quality and accuracy, as mentioned earlier, DATASUS is a database managed by the government of Brazil that consolidates information from both the public and private sectors. However, the accuracy of the data in this database is dependent on the accurate input of information by health professionals, which may lead to potential inaccuracies. Furthermore, the dataset provided by DATASUS does not include crucial patient details such as demographics, comorbidities, surgery complications, and causes of mortality during hospitalization. In addition, there may be other concomitant illnesses, or comorbidities that may have accelerated patient death after performing a specific surgery that were not specified as the initial cause of death. 

The use of CFR as a marker for surgical mortality has some limitations, as many confounding variables may not be identifiable from the data. CFR calculations may not fully adjust for pre-existing conditions or comorbidities that can significantly impact surgical outcomes. Patients with multiple health issues may have higher mortality rates, which might not be entirely reflected in CFR calculation. Additionally, CFR may not consider variations in case severity among different patient populations or types of surgical procedures. Some surgeries inherently carry higher risks, and patients with more complex medical conditions may have higher mortality rates regardless of the specific surgical intervention. Furthermore, the outcomes of surgeries and the number of readmissions are not explored in this study because the nature of our study does not imply such discussion because it’s a trend analysis. However, it is an interesting area that requires further exploration in future studies.

Despite these limitations, our research is a novel and comprehensive study. It displays a description of 4 distinct periods of the COVID-19 pandemic. Furthermore, data was allocated for multiple surgical procedures and we utilized a more thorough and convenient statistical approach to reach more reliable inferences.

## Conclusions

The novel coronavirus SARS-CoV-2 pandemic had a profound impact on case fatality rates and the cost of surgical care in Brazil. Analysis of costs of procedures according to subgroups revealed a decreasing pattern during the pandemic for most of the surgeries, with a gradual return to baseline values during the recovery and post-pandemic periods except for the thoracic surgery which revealed a higher cost during the recovery period. Furthermore, case fatality rates across the regions had a pattern in which the rates initially increased during the pandemic followed by a gradual decline in the recovery period, and only to subsequently drop reaching the baseline during the postoperative period. The pattern was similar when analyzing the case fatality rates and ratios according to subgroups. The benefits of our study are not only limited to the Brazilian population. The results inferred from our study can be adopted by the policymakers of other countries as well, especially by the upper-middle income countries like Brazil. In addition, this study recommends performing similar studies in the future in other parts of the world especially in developing countries to further strengthen our results. Also, it requires exploring other areas like the impact of COVID-19 on surgical outcomes in terms of readmission and development of comorbidities. Hence, we conclude that the challenges caused by the pandemic affected the country’s healthcare system, leading to significant challenges in providing medical services, including surgical care.
